# 1,1′-[(1*E*,11*E*)-5,8-Dioxa-2,11-diazo­nia­dodeca-1,11-diene-1,12-di­yl]dinaph­thal­en-2-olate

**DOI:** 10.1107/S1600536811012219

**Published:** 2011-04-07

**Authors:** Yanju Liu, Kai Liu, Zhiqiang Cao, Meiju Niu

**Affiliations:** a State Key Laboratory of Water Environment Simulation, School of Environment, Beijing Normal University, Beijing 100875, People’s Republic of China; b Dongchang College, Liaocheng University, Shandong 252059, People’s Republic of China; c College of Chemistry and Chemical Engineering, Liaocheng University, Shandong 252059, People’s Republic of China

## Abstract

The title compound, C_28_H_28_N_2_O_4_, crystallizes in a zwitterionic form with deprotonated naphthol hy­droxy groups and protonated imine N atoms. The asymmetric unit contains one half-mol­ecule located on a twofold rotation axis. Intra­molecular N—H⋯O hydrogen bonds occur and the two bicyclic ring systems form a dihedral angle of 64.2 (1)°. In the crystal, weak inter­molecular C—H⋯O hydrogen bonds link the mol­ecules into layers parallel to the *bc* plane.

## Related literature

For applications of Schiff bases in coordination chemistry, see: Osowle (2008 ▶). For related structures, see: Etemadi *et al.* (2004 ▶); Liu *et al.* (2010 ▶); Farag *et al.* (2010 ▶; 2011 ▶).
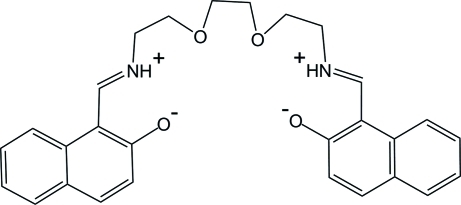


## Experimental

### 

#### Crystal data

C_28_H_28_N_2_O_4_
*M* *_r_* = 456.52
Orthorhombic, 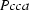

*a* = 44.704 (4) Å
*b* = 6.3576 (6) Å
*c* = 8.2074 (9) Å
*V* = 2332.6 (4) Å^3^
*Z* = 4
Mo *K*α radiation
μ = 0.09 mm^−1^
*T* = 298 K
0.50 × 0.37 × 0.11 mm

#### Data collection

Bruker SMART APEX CCD area-detector diffractometer
Absorption correction: multi-scan (*SADABS*; Bruker, 2009 ▶) *T*_min_ = 0.958, *T*_max_ = 0.991
9099 measured reflections
2060 independent reflections
1162 reflections with *I* > 2σ(*I*)
*R*_int_ = 0.081

#### Refinement

*R*[*F* ^2^ > 2σ(*F* ^2^)] = 0.068
*wR*(*F* ^2^) = 0.224
*S* = 1.00
2060 reflections
155 parameters
H-atom parameters constrained
Δρ_max_ = 0.21 e Å^−3^
Δρ_min_ = −0.19 e Å^−3^

### 

Data collection: *APEX2* (Bruker, 2009 ▶); cell refinement: *SAINT* (Bruker, 2009 ▶); data reduction: *SAINT*; program(s) used to solve structure: *SHELXTL* (Sheldrick, 2008 ▶); program(s) used to refine structure: *SHELXTL*; molecular graphics: *SHELXTL*; software used to prepare material for publication: *SHELXTL*.

## Supplementary Material

Crystal structure: contains datablocks I, global. DOI: 10.1107/S1600536811012219/cv5066sup1.cif

Structure factors: contains datablocks I. DOI: 10.1107/S1600536811012219/cv5066Isup2.hkl

Additional supplementary materials:  crystallographic information; 3D view; checkCIF report

## Figures and Tables

**Table 1:** Hydrogen-bond geometry (Å, °)

*D*—H⋯*A*	*D*—H	H⋯*A*	*D*⋯*A*	*D*—H⋯*A*
N1—H1*A*⋯O1	0.86	1.85	2.542 (5)	136
C14—H14*B*⋯O2^i^	0.97	2.58	3.382 (5)	141
C12—H12*B*⋯O1^ii^	0.97	2.50	3.257 (5)	134

Symmetry codes: (i) 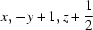; (ii) 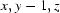.

**Fig. 1.**
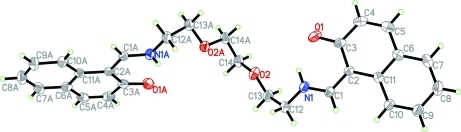
The molecular structure of (I) showing the atomic labels and 30% probability displacement ellipsoids. Symmetry code: (A) -x + 1/2,-y + 1,z.

## supplementary crystallographic information

### Comment

Schiff bases have various applications in coordination chemistry (Osowle, 2008). Herewith we present the title compound (I), which is a new crowned Schiff base.

In (I) (Fig. 1), all bond lengths and angles are usual and comparable with those observed in the related compounds (Etemadi *et al.*, 2004; Liu *et al.*, 2010; Farag *et al.*, 2010, 2011). Each molecule is situated on a twofold rotational axis. Intramolecular N—H···O hydrogen bonds (Table 1) influence the molecular conformation - two bicycles form a dihedral angle of 64.2 (1)°. In the crystal structure, weak intermolecular C—H···O hydrogen bonds (Table 1) link molecules into layers parallel to *bc* plane.

### Experimental

The title compound was synthesized by adding drop-wise a solution of 3,6-dioxa-1,8-diaminooctane (1.48 g,10 mmol) in absolute methanol (10 mL) to a methanol solution (20 mL) of 2-hydroxy-1-naphthaldehyde (3.4438 g,20 mmol) under stirring at room temperature. The resultant reaction mixture was then refluxed for 5 h, cooled and concentrated under reduced pressure, and then the residue was retained at -268 K for overnight. The bright yellow crystal which suitable for X-ray analysis was formed, filtered and dried under reduced pressure.Yield:82%. Analysis found: C 73.07, H 6.06, N 6.51%; calculated for C_28_H_28_N_2_O_4_ (Mr=456.54): C 73.66, H 6.18, N 6.14%.

### Refinement

C-bound H atoms were geometrically positioned [C—H 0.93–0.97 Å]. Atom H1A was located on a difference map, but placed in idealized position [N—H 0.86 Å]. All H atoms were refined as riding atoms, with *U*_iso_(H) = 1.2–1.5 *U*_iso_ of the parent atom.

### Crystal data

**Table:**

C_28_H_28_N_2_O_4_	*F*(000) = 968
*M**_r_* = 456.52	*D*_x_ = 1.300 Mg m^−^^3^
Orthorhombic, *P**c**c**a*	Mo *K*α radiation, λ = 0.71073 Å
Hall symbol: -P 2a 2ac	Cell parameters from 1398 reflections
*a* = 44.704 (4) Å	θ = 2.7–22.6°
*b* = 6.3576 (6) Å	µ = 0.09 mm^−^^1^
*c* = 8.2074 (9) Å	*T* = 298 K
*V* = 2332.6 (4) Å^3^	Block, yellow
*Z* = 4	0.50 × 0.37 × 0.11 mm

### Data collection

**Table:**

Bruker SMART APEX CCD area-detector diffractometer	2060 independent reflections
Radiation source: fine-focus sealed tube	1162 reflections with *I* > 2σ(*I*)
graphite	*R*_int_ = 0.081
phi and ω scans	θ_max_ = 25.0°, θ_min_ = 1.8°
Absorption correction: multi-scan (*SADABS*; Bruker, 2009)	*h* = −53→28
*T*_min_ = 0.958, *T*_max_ = 0.991	*k* = −7→7
9099 measured reflections	*l* = −9→9

### Refinement

**Table:**

Refinement on *F*^2^	Secondary atom site location: difference Fourier map
Least-squares matrix: full	Hydrogen site location: inferred from neighbouring sites
*R*[*F*^2^ > 2σ(*F*^2^)] = 0.068	H-atom parameters constrained
*wR*(*F*^2^) = 0.224	*w* = 1/[σ^2^(*F*_o_^2^) + (0.088*P*)^2^ + 3.8548*P*] where *P* = (*F*_o_^2^ + 2*F*_c_^2^)/3
*S* = 1.00	(Δ/σ)_max_ < 0.001
2060 reflections	Δρ_max_ = 0.21 e Å^−^^3^
155 parameters	Δρ_min_ = −0.19 e Å^−^^3^
0 restraints	Extinction correction: *SHELXL*, Fc^*^=kFc[1+0.001xFc^2^λ^3^/sin(2θ)]^-1/4^
Primary atom site location: structure-invariant direct methods	Extinction coefficient: 0.0055 (16)

### Fractional atomic coordinates and isotropic or equivalent isotropic displacement parameters (Å^2^)

**Table:**

	*x*	*y*	*z*	*U*_iso_*/*U*_eq_	
N1	0.16369 (7)	0.4462 (5)	0.0795 (4)	0.0479 (10)	
H1A	0.1720	0.5537	0.1239	0.058*	
C2	0.11853 (8)	0.6454 (6)	0.0728 (5)	0.0370 (9)	
O1	0.15974 (7)	0.8123 (5)	0.2021 (4)	0.0628 (10)	
O2	0.22075 (6)	0.4082 (5)	0.2116 (4)	0.0541 (9)	
C11	0.08741 (8)	0.6581 (6)	0.0253 (5)	0.0388 (10)	
C1	0.13580 (8)	0.4646 (6)	0.0400 (5)	0.0402 (10)	
H1	0.1266	0.3525	−0.0129	0.048*	
C6	0.07056 (9)	0.8373 (6)	0.0703 (5)	0.0464 (11)	
C3	0.13216 (10)	0.8124 (6)	0.1618 (5)	0.0463 (11)	
C10	0.07267 (8)	0.5045 (7)	−0.0672 (5)	0.0469 (11)	
H10	0.0832	0.3861	−0.1007	0.056*	
C12	0.18209 (9)	0.2620 (7)	0.0560 (6)	0.0505 (12)	
H12A	0.1941	0.2793	−0.0415	0.061*	
H12B	0.1694	0.1397	0.0410	0.061*	
C4	0.11373 (10)	0.9880 (7)	0.2060 (6)	0.0557 (12)	
H4	0.1220	1.0978	0.2657	0.067*	
C5	0.08502 (10)	0.9976 (7)	0.1636 (5)	0.0550 (12)	
H5	0.0739	1.1138	0.1962	0.066*	
C8	0.02674 (10)	0.6990 (9)	−0.0632 (6)	0.0650 (14)	
H8	0.0067	0.7120	−0.0922	0.078*	
C7	0.04054 (10)	0.8504 (8)	0.0252 (6)	0.0572 (13)	
H7	0.0296	0.9677	0.0571	0.069*	
C9	0.04327 (9)	0.5238 (8)	−0.1096 (6)	0.0553 (12)	
H9	0.0342	0.4182	−0.1703	0.066*	
C13	0.20210 (9)	0.2282 (7)	0.1994 (6)	0.0566 (13)	
H13A	0.1903	0.2115	0.2979	0.068*	
H13B	0.2141	0.1027	0.1840	0.068*	
C14	0.24105 (8)	0.4015 (7)	0.3430 (5)	0.0507 (12)	
H14A	0.2542	0.2808	0.3319	0.061*	
H14B	0.2302	0.3884	0.4449	0.061*	

### Atomic displacement parameters (Å^2^)

**Table:**

	*U*^11^	*U*^22^	*U*^33^	*U*^12^	*U*^13^	*U*^23^
N1	0.0378 (17)	0.048 (2)	0.059 (2)	−0.0032 (15)	−0.0074 (17)	0.0003 (19)
C2	0.041 (2)	0.035 (2)	0.035 (2)	−0.0033 (17)	−0.0019 (18)	0.0031 (18)
O1	0.066 (2)	0.0485 (19)	0.074 (2)	−0.0125 (15)	−0.0256 (18)	0.0002 (17)
O2	0.0413 (15)	0.064 (2)	0.057 (2)	−0.0092 (14)	−0.0097 (14)	0.0128 (16)
C11	0.042 (2)	0.042 (2)	0.032 (2)	0.0007 (18)	0.0030 (18)	0.0051 (19)
C1	0.039 (2)	0.042 (2)	0.040 (2)	−0.0059 (17)	−0.0017 (19)	0.0059 (19)
C6	0.054 (2)	0.043 (2)	0.043 (3)	0.000 (2)	0.007 (2)	0.004 (2)
C3	0.059 (3)	0.037 (2)	0.043 (3)	−0.006 (2)	−0.005 (2)	0.004 (2)
C10	0.043 (2)	0.050 (2)	0.049 (3)	−0.003 (2)	−0.004 (2)	−0.003 (2)
C12	0.040 (2)	0.054 (3)	0.057 (3)	0.000 (2)	−0.003 (2)	−0.003 (2)
C4	0.081 (3)	0.041 (2)	0.046 (3)	−0.011 (3)	−0.004 (3)	−0.002 (2)
C5	0.074 (3)	0.043 (3)	0.049 (3)	0.006 (2)	0.006 (2)	0.001 (2)
C8	0.040 (2)	0.091 (4)	0.064 (3)	0.005 (3)	−0.001 (2)	0.001 (3)
C7	0.052 (3)	0.061 (3)	0.059 (3)	0.017 (2)	0.008 (2)	0.005 (3)
C9	0.045 (2)	0.068 (3)	0.053 (3)	−0.001 (2)	−0.006 (2)	−0.005 (3)
C13	0.047 (2)	0.054 (3)	0.069 (3)	0.000 (2)	−0.011 (2)	0.004 (3)
C14	0.044 (2)	0.067 (3)	0.042 (3)	0.004 (2)	−0.003 (2)	0.005 (2)

### Geometric parameters (Å, °)

**Table:**

N1—C1	1.294 (4)	C12—C13	1.494 (6)
N1—C12	1.444 (5)	C12—H12A	0.9700
N1—H1A	0.8600	C12—H12B	0.9700
C2—C1	1.410 (5)	C4—C5	1.331 (6)
C2—C3	1.426 (5)	C4—H4	0.9300
C2—C11	1.447 (5)	C5—H5	0.9300
O1—C3	1.276 (5)	C8—C7	1.354 (7)
O2—C14	1.410 (5)	C8—C9	1.390 (6)
O2—C13	1.420 (5)	C8—H8	0.9300
C11—C10	1.402 (5)	C7—H7	0.9300
C11—C6	1.415 (5)	C9—H9	0.9300
C1—H1	0.9300	C13—H13A	0.9700
C6—C7	1.394 (6)	C13—H13B	0.9700
C6—C5	1.429 (6)	C14—C14^i^	1.486 (8)
C3—C4	1.434 (6)	C14—H14A	0.9700
C10—C9	1.365 (5)	C14—H14B	0.9700
C10—H10	0.9300		
			
C1—N1—C12	126.1 (4)	C5—C4—C3	121.6 (4)
C1—N1—H1A	117.0	C5—C4—H4	119.2
C12—N1—H1A	117.0	C3—C4—H4	119.2
C1—C2—C3	118.1 (3)	C4—C5—C6	122.9 (4)
C1—C2—C11	121.4 (3)	C4—C5—H5	118.6
C3—C2—C11	120.5 (4)	C6—C5—H5	118.6
C14—O2—C13	114.1 (3)	C7—C8—C9	118.3 (4)
C10—C11—C6	116.9 (4)	C7—C8—H8	120.8
C10—C11—C2	124.0 (4)	C9—C8—H8	120.8
C6—C11—C2	119.1 (4)	C8—C7—C6	122.6 (4)
N1—C1—C2	123.6 (4)	C8—C7—H7	118.7
N1—C1—H1	118.2	C6—C7—H7	118.7
C2—C1—H1	118.2	C10—C9—C8	120.9 (5)
C7—C6—C11	119.4 (4)	C10—C9—H9	119.5
C7—C6—C5	122.3 (4)	C8—C9—H9	119.5
C11—C6—C5	118.2 (4)	O2—C13—C12	106.9 (4)
O1—C3—C2	123.1 (4)	O2—C13—H13A	110.3
O1—C3—C4	119.3 (4)	C12—C13—H13A	110.3
C2—C3—C4	117.6 (4)	O2—C13—H13B	110.3
C9—C10—C11	121.9 (4)	C12—C13—H13B	110.3
C9—C10—H10	119.1	H13A—C13—H13B	108.6
C11—C10—H10	119.1	O2—C14—C14^i^	108.7 (3)
N1—C12—C13	110.7 (4)	O2—C14—H14A	109.9
N1—C12—H12A	109.5	C14^i^—C14—H14A	109.9
C13—C12—H12A	109.5	O2—C14—H14B	109.9
N1—C12—H12B	109.5	C14^i^—C14—H14B	109.9
C13—C12—H12B	109.5	H14A—C14—H14B	108.3
H12A—C12—H12B	108.1		
			
C1—C2—C11—C10	−5.6 (6)	C2—C11—C10—C9	−179.8 (4)
C3—C2—C11—C10	177.6 (4)	C1—N1—C12—C13	−140.1 (4)
C1—C2—C11—C6	175.9 (4)	O1—C3—C4—C5	178.5 (4)
C3—C2—C11—C6	−0.9 (6)	C2—C3—C4—C5	−1.1 (7)
C12—N1—C1—C2	177.0 (4)	C3—C4—C5—C6	−0.8 (7)
C3—C2—C1—N1	−3.4 (6)	C7—C6—C5—C4	−179.3 (5)
C11—C2—C1—N1	179.8 (4)	C11—C6—C5—C4	1.8 (7)
C10—C11—C6—C7	1.5 (6)	C9—C8—C7—C6	0.3 (7)
C2—C11—C6—C7	−179.9 (4)	C11—C6—C7—C8	−1.1 (7)
C10—C11—C6—C5	−179.5 (4)	C5—C6—C7—C8	180.0 (4)
C2—C11—C6—C5	−0.9 (6)	C11—C10—C9—C8	0.5 (7)
C1—C2—C3—O1	5.5 (6)	C7—C8—C9—C10	0.0 (7)
C11—C2—C3—O1	−177.7 (4)	C14—O2—C13—C12	179.6 (3)
C1—C2—C3—C4	−175.0 (4)	N1—C12—C13—O2	−61.2 (5)
C11—C2—C3—C4	1.8 (6)	C13—O2—C14—C14^i^	−179.5 (4)
C6—C11—C10—C9	−1.3 (6)		

Symmetry codes: (i) −*x*+1/2, −*y*+1, *z*.

### Hydrogen-bond geometry (Å, °)

**Table:**

*D*—H···*A*	*D*—H	H···*A*	*D*···*A*	*D*—H···*A*
N1—H1A···O1	0.86	1.85	2.542 (5)	136
C14—H14B···O2^ii^	0.97	2.58	3.382 (5)	141
C12—H12B···O1^iii^	0.97	2.50	3.257 (5)	134

Symmetry codes: (ii) *x*, −*y*+1, *z*+1/2; (iii) *x*, *y*−1, *z*.
